# Self-assembled nanoparticles containing photosensitizer and polycationic brush for synergistic photothermal and photodynamic therapy against periodontitis

**DOI:** 10.1186/s12951-021-01114-w

**Published:** 2021-12-11

**Authors:** Enyu Shi, Liya Bai, Lujia Mao, Hanping Wang, Xiaoying Yang, Yinsong Wang, Mingming Zhang, Changyi Li, Yue Wang

**Affiliations:** 1 School of Dentistry & Hospital of Stomatology, Tianjin Medical University, Tianjin, 300070 China; 2 Tianjin Key Laboratory of Technologies Enabling Development of Clinical Therapeutics and Diagnostics, School of Pharmacy, Tianjin Medical University, Tianjin, 300070 China; 3 Tianjin Key Laboratory of Biomedical Materials, Institute of Biomedical Engineering, Chinese Academy of Medical Sciences & Peking Union Medical College, Tianjin, 300192 China

**Keywords:** Nanoparticles, Polycationic brush, Photothermal therapy, Photodynamic therapy, Periodontitis

## Abstract

**Background:**

Periodontitis is a chronic inflammatory disease in oral cavity owing to bacterial infection. Photothermal therapy (PTT) and photodynamic therapy (PDT) have many advantages for antibacterial treatment. As an excellent photosensitizer, indocyanine green (ICG) shows prominent photothermal and photodynamic performances. However, it is difficult to pass through the negatively charged bacterial cell membrane, thus limiting its antibacterial application for periodontitis treatment.

**Results:**

In this work, self-assembled nanoparticles containing ICG and polycationic brush were prepared for synergistic PTT and PDT against periodontitis. First, a star-shaped polycationic brush poly(2-(dimethylamino)ethyl methacrylate) (sPDMA) was synthesized via atom transfer radical polymerization (ATRP) of DMA monomer from bromo-substituted β-cyclodextrin initiator (CD-Br). Next, ICG was assembled with sPDMA to prepare ICG-loaded sPDMA (sPDMA@ICG) nanoparticles (NPs) and the physicochemical properties of these NPs were characterized systematically. In vitro antibacterial effects of sPDMA@ICG NPs were investigated in porphyromonas gingivalis (Pg), one of the recognized periodontitis pathogens. A ligature-induced periodontitis model was established in Sprague–Dawley rats for in vivo evaluation of anti-periodontitis effects of sPDMA@ICG NPs. Benefiting from the unique brush-shaped architecture of sPDMA polycation, sPDMA@ICG NPs significantly promoted the adsorption and penetration of ICG into the bacterial cells and showed excellent PTT and PDT performances. Both in vitro and in vivo, sPDMA@ICG NPs exerted antibacterial and anti-periodontitis actions via synergistic PTT and PDT.

**Conclusions:**

A self-assembled nanosystem containing ICG and polycationic brush has shown promising clinical application for synergistic PTT and PDT against periodontitis.

**Graphical Abstract:**

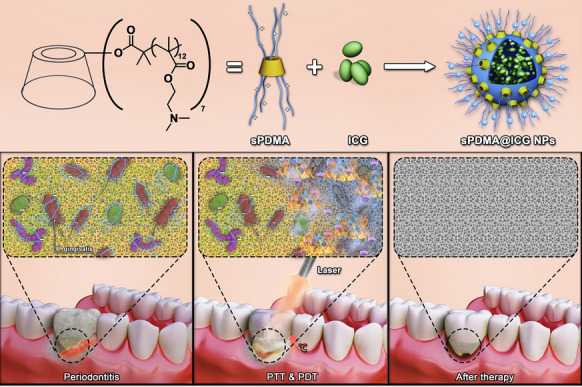

**Supplementary Information:**

The online version contains supplementary material available at 10.1186/s12951-021-01114-w.

## Introduction

Periodontitis is the sixth most prevalent disease in the world, with more than 743 million people worldwide at risk [1]. It is a chronic inflammatory disease caused by bacteria, which eventually leads to the formation of periodontal pockets, the loss of alveolar bone and the loosening of teeth [2, 3]. In addition, periodontal disease is reported to be related to some systemic diseases, such as arteriosclerosis, diabetes, aspiration pneumonia and vascular disease [4]. At present, mechanical debridement and antibiotics are commonly used in clinic [5, 6]. However, in most cases, mechanical debridement is difficult to completely remove the periodontitis infection in deep periodontal pocket, furcation and irregular area of root surface [7, 8]. Antibiotic treatment can effectively eliminate periodontal pathogens, but long-term use of antibiotics will bring many problems, such as producing drug-resistant bacteria (so-called superbacteria), dysbacteriosis, gastrointestinal diseases and so on [9, 10]. Hence, it is necessary to develop a more effective method for periodontitis treatment.

Phototherapy, including photothermal therapy (PTT) and photodynamic therapy (PDT), is widely recognized as an excellent non-invasive approach for cancer ablation [11–13] and also has been used for antimicrobial treatment in recent years [14, 15]. PTT is a hyperthermia therapeutic method that applies near-infrared (NIR) light-absorbing agents to exert a lethal temperature elevation effect on bacteria under laser irradiation [16]. PDT can induce the generation of cytotoxic reactive oxygen species (ROS) to bring about the fatal and extensive cell damages through the oxidation of nucleic acids, lipids and proteins [17, 18], based on the photochemical reaction that involves three basic components (photosensitizer, molecular oxygen and laser at a particular wavelength). These phototherapeutic methods have a high spatiotemporal selectivity for localized tissue ablation, and thus are highly safe for applications in vivo [19]. More importantly, they are equally effective to drug-resistant and non-drug-resistant pathogenic bacteria, and will not cause bacterial resistance even if a long-term use [20]. An increasing number of investigations have been carried out for antimicrobial treatment through PTT and PDT. In a recent report [21], we developed a novel photosensitizer with excellent bacteria penetrating ability and combined PDT and antibiotics for periodontitis treatment by fighting periodontal pathogenic bacteria more efficiently.

Some NIR light-absorbing agents exhibit prominent photothermal and photodynamic performances simultaneously. Indocyanine green (ICG) is a highly safe NIR light-absorbing agent and has been approved by the Food and Drug Administration (FDA) for clinical use since 1959. In recent decade, it has been widely applied for cancer treatment via synergistic PTT and PDT [22]. However, because of its negative charges and water solubility, ICG is difficult to pass through the bacterial cell membrane that is also negatively charged and characterized by lipid bimolecular structure. Gram-negative bacteria have the inner and the outer membranes composed of phospholipids and lipopolysaccharide, and thus it is more difficult for ICG to penetrate both these bacterial membranes. In addition, as a natural barrier from external dangers [23], the bacterial biofilm further prevents the penetration of ICG. All these limitations will be unfavorable for fully exerting the PTT and PDT performances of ICG against pathogenic bacteria.

Some cationic polymers exhibit strong bacterial cell penetration activity, mainly manifested their adsorption onto the bacterial surfaces with negative charges [24, 25], and even interaction with the inner membranes of gram-negative bacteria [26]. Polycationic molecular brushes are a novel kind of branched cationic polymers that is defined as dense layers of cationic polymer chains grafted on a molecule. Compared to the linear or ordinary branched cationic polymers, when a polymer is in brush region, the distance (D) between the neighboring chains is less than twice of gyration radius (R_g_) of a free polymer chain (D/R_g_ < 2). With such high grafting density, the chains are forced to stretch out due to electrostatic repulsion and the geometrical constraint. And therefore, the stability of carriers constructed by the brush can be improved due to the high osmotic repulsion within the brush layer. Besides, the densely positive chains endow polycationic brushes significant binding ability with the negatively charged cell membranes, resulting in the decrease of their working concentrations. Polycationic brushes are excellent gene carriers and often exhibit higher gene transfection efficiencies [27–29] and less cytotoxicity than the linear ones with comparable molecular weights [30]. Consideration of the above-mentioned advantages, we believe that polycationic brushes can be used as a novel kind of carrier materials for antibacterial agents.

**Scheme 1.**
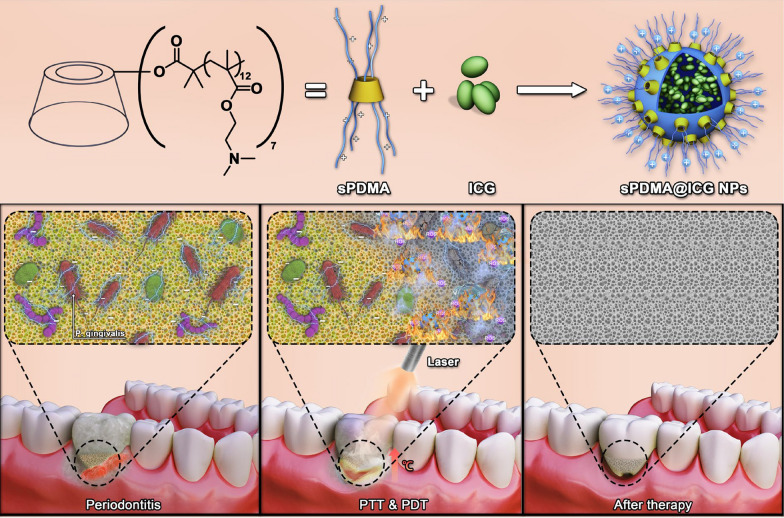
Illustrations for preparation of sPDMA@ICG NPs and treatment progress of synergistic PTT and PDT on periodontitis

In this study, a self-assembled nanosystem of ICG and polycationic brushes was developed for synergistic PTT and PDT against periodontitis (Scheme 1). First, a star-shaped polycationic brush poly(2-(dimethylamino)ethyl methacrylate) (sPDMA) was synthesized via atom transfer radical polymerization (ATRP) of DMA monomer from bromo-substituted β-cyclodextrin initiator (CD-Br). Next, ICG was assembled with sPDMA to prepare ICG-loaded sPDMA (sPDMA@ICG) nanoparticles (NPs) and the physicochemical properties of these NPs were characterized systematically. In vitro antibacterial effects of sPDMA@ICG NPs were investigated in porphyromonas gingivalis (Pg) [31, 32], one of the recognized periodontitis pathogens. A ligature-induced periodontitis model was established in Sprague–Dawley rats for in vivo evaluation of anti-periodontitis effects of sPDMA@ICG NPs. Benefiting from the unique brush-shaped architecture of sPDMA polycation, sPDMA@ICG NPs significantly promoted the adsorption and penetration of ICG into the bacterial cells and showed excellent PTT and PDT performances. Both in vitro and in vivo, sPDMA@ICG NPs exerted effective antibacterial and anti-periodontitis actions via synergistic PTT and PDT.

## Materials and methods

### Materials

ICG was purchased from J&K Scientific Ltd. (Beijing, China). 2-(Dimethylamino) ethyl methacrylate (DMA) and 2-bromoisobutyryl bromide (BiBB) both with a purity of approximately 98% were bought from Sigma-Aldrich (Milwaukee, USA), and DMA was passed through a basic alumina column before use to remove polymerization inhibitor. β-Cyclodextrin (β-CD) and 2,2-bipyridine (Bpy) were supplied by Sinopharm Chemical Regent Co., Ltd. (Shanghai, China). Anhydrous N-methyl-2-pyrrolidone (NMP) was obtained from Alfa Aesar (Ward Hill, MA, USA). Cuprous chloride (CuCl) was dissolved in concentrated hydrochloric acid, precipitated via dilution with water, washed separately with ethanol and ethyl ether, and finally vacuum dried in an oven. Copper chloride (CuCl_2_) was baked at 120 °C to remove the crystal water before use. 2,7-Dichlorodihydro fluorescein diacetate (DCFH-DA), N-phenyl-1-naphthylamine (NPN), 4', 6-diamidine-2-phenylindole dihydrochloride (DAPI), and hematoxylin & eosin (H&E) were all purchased from Sigma-Aldrich. Singlet Oxygen Sensor Green Reagent (SOSG), Cell Counting Kit-8 (CCK-8), and the LIVE/DEAD BacLight Bacterial Viability Kit were purchased from Thermo Fisher Scientific (Waltham, MA, USA).

### Cells and animals

The strain of Pg (code BNCC 337,441), sourced from BeNa Culture Collection (Beijing, China), was cultured in the Brain Heart Infusion (BHI) Broth or the Columbia blood agar plates (Solarbio, Beijing, China) at 37 °C in an anaerobic chamber with an atmosphere of 80% N_2_, 10% H_2_ and 10% CO_2_.

Female Sprague Dawley (SD) rats with average body weight of 150 g were provided by Vital River Laboratory Animal Technology Co., Ltd. (Beijing, China) and were kept in plastic cages with free access to food and water. Periodontitis animal model was constructed in SD rats according to our previous method [17]. After anesthetization, the rats were ligated with orthodontic steel wires at the gingival sulcus of the left maxillary second molar and then feed with 10% sucrose water for 4 weeks to induce periodontitis. All animal experiments in this study were carried out according to the protocol approved by the Tianjin Medical University Animal Care and Use Committee.

### Preparation and characterization of sPDMA@ICG NPs

CD-Br was firstly synthesized via the esterification of β-CD with BiBB according to the previous work [32] and then polycationic brush sPDMA was synthesized via atom transfer radical polymerization (ATRP) of DMA monomer using CD-Br as an initiator [33]. The detailed methods were described in the supporting information.

Next, an improved nanoprecipitation method was used to prepare sPDMA@ICG NPs. Briefly, ICG (5 mg) was dissolved in 100 μL of dimethyl sulfoxide (DMSO) as a stock solution, and meanwhile 14 mg of sPDMA was dissolved in 10 mL deionized water. After that, 2, 4, 8, 12, and 26 μL of ICG stock solution were mixed separately with 1 mL of sPDMA aqueous solution and stirred for 12 h at room temperature in the dark, thus obtaining sPDMA@ICG NPs with different ICG contents. The ultraviolet–visible-near infrared (UV–Vis-NIR) absorption spectra of these sPDMA@ICG NPs were recorded under a U-3310 spectrophotometer (Hitachi High-Tech, Tokyo, Japan). Here, 8 μL of ICG stock solution was diluted with 1 mL of deionized water to be used as the control. The diameters and polydispersity indexes (PDIs) of these NPs were detected using an automatic particle size detector (Zetasizer Nano ZS, Malvern, UK).

By comprehensive consideration, the ICG/sPDMA weight ratio of 4/14 was chosen to prepare sPDMA@ICG NPs using the same method as above. The thus-obtained nanoparticle dispersion was transferred into a dialysis bag (MWCO 500 Da) and dialyzed against deionized water to remove DMSO and unassembled ICG. Finally, the morphology of sPDMA@ICG NPs was characterized under a HT7700 transmission electron microscope (TEM) (Hitachi High-Tech, Tokyo, Japan) and their size and size distribution were further detected.

### Statistical analysis

All the experiments were carried out independently at least three times. The experimental data were presented as mean ± standard deviation (SD). Statistical analysis was performed by one-way analysis of variance (ANOVA) and P < 0.05 was considered to be significant statistically.

## Results and discussion

### Preparation and characterization of sPDMA@ICG NPs

**Fig. 1:**
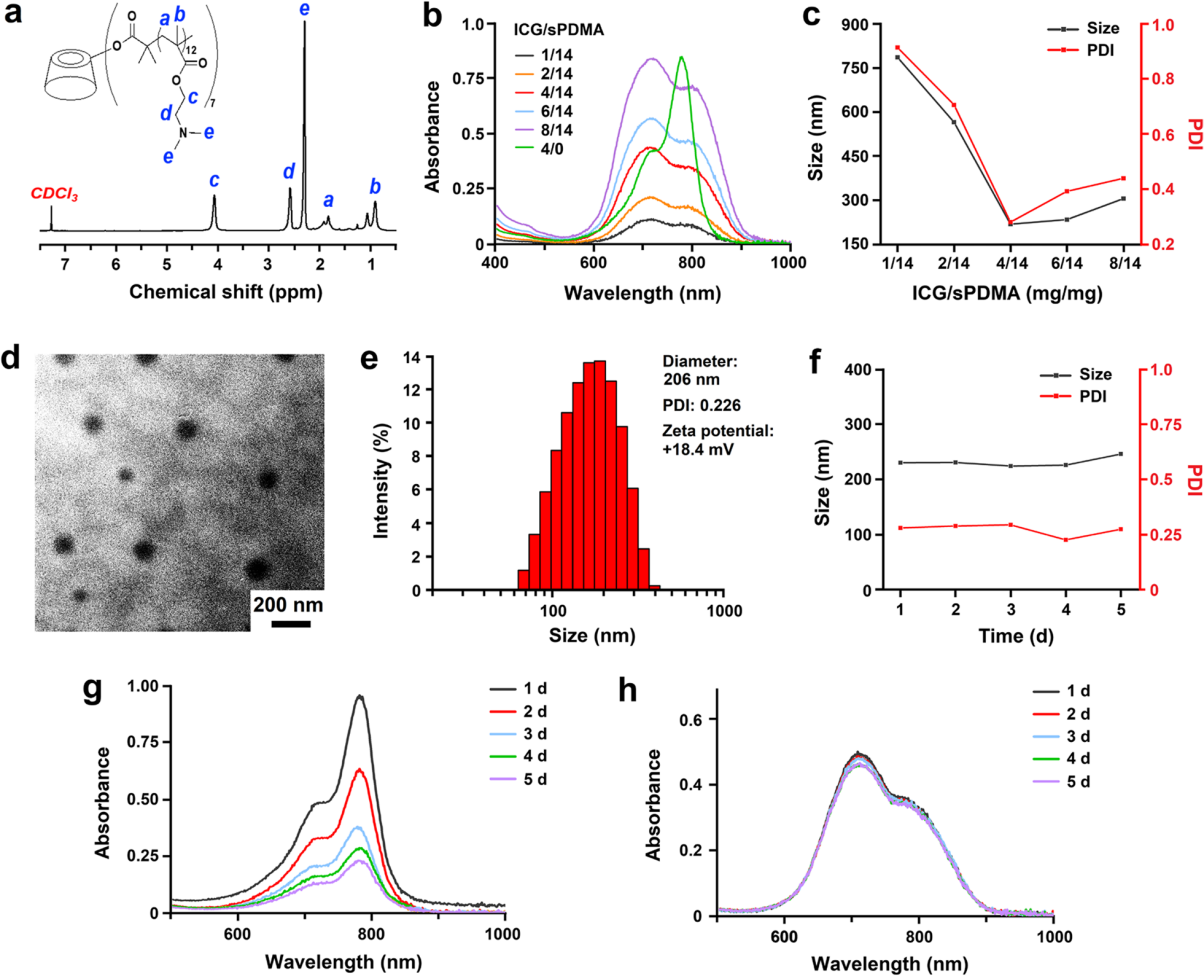
Preparation and characterization of sPDMA@ICG NPs. **a** Chemical structure and ^1^H NMR spectrum of sPDMA in CDCl_3_. **b** UV–Vis-NIR absorption spectra, **c** size and PDI of sPDMA@ICG NPs prepared at different weight ratios of ICG/sPDMA. **d** TEM image and **e** size distribution of sPDMA@ICG NPs at the ICG/sPDMA weight ratio of 4/14. **f** Sizes and PDIs of sPDMA@ICG NPs during storage for 5 d. UV–Vis-NIR adsorption spectra of **g** free ICG and **h** sPDMA@ICG NPs in aqueous solution during storage for 5 d at the ICG concentration of 10 μg/mL

A star-shaped polycation sPDMA was first synthesized via ATRP of DMA monomer from CD-Br initiator according to our previous works [32], and the synthesis route is shown in Additional file 1: Fig. S1. From the ^1^H NMR spectrum of CD-Br initiator in Additional file 1: Fig. S2, the number of Br per β-CD molecule was calculated to be 7, indicating that 7 PDMA chains were grafted from one β-CD. After polymerization, the characteristic peaks representing protons in PDMA appeared at 4.1, 2.6 and 2.3 ppm (Fig. 1a), proving the success of polymerization. Since the content of β-CD in the polymer was relatively small, the proton signals of β-CD were not observed in the spectrum. According to the polymerization yield, the degree of polymerization (D_p_) was estimated to be about 12.2 per chain and the molecular weight of sPDMA was approximately 15,600. The molecular weight distribution (M_w_/M_n_) was 1.24 characterized by gel permeation chromatography. To validate whether the polymer is in molecular brush regime, the gyration radius (R_g_) and the distance (D) between the neighboring chains were estimated. According to the equation (R_g_ = 0.5D_p_^0.5^) [34], R_g_ of PDMA chain was calculated to be about 1.75 nm, while D was estimated to be less than the outer diameter of β-CD (1.54 nm) [35]. Hence, D/R_g_ would be less than 0.88, meaning that the PDMA chains are in molecular brush regime. With such high grafting density, the PDMA chains will be forced into a stretched state due to the steric constraint, endowing sPDMA unique properties different from its random coil state.

sPDMA@ICG NPs were prepared from self-assembly of ICG and sPDMA in aqueous media using the nanoprecipitation method described in the Supporting information. The electronic interaction and inclusion effect played important roles in the self-assembly between ICG and sPDMA. First, ICG and sPDMA have positive and negative charges separately and hence the electrostatic interaction exists between their molecules. Second, ICG has hydrophobic molecular character and can be partially included into the cavity of β-cyclodextrin grafted on sPDMA via hydrophobic interaction. This was evidenced by the ultraviolet–visible-near infrared (UV–Vis-NIR) absorption spectra in Additional file 1: Fig. S3. After assembled with sPDMA, ICG showed an obvious red shift in the characteristic absorption peak, indicating that ICG was located in a less polar microenvironment. Besides, the peak intensity of ICG monomer at 780 nm significantly decreased, while that of ICG dimer at 700 nm increased a little at the same time. This demonstrated that the distance between ICG molecules is closer in the NPs than in aqueous solution according to a previous report [36].

sPDMA@ICG NPs with different weight ratios of ICG/sPDMA were further prepared using the same method. The similar peak shapes were observed in the UV–Vis-NIR spectra of these NPs (Fig. 1b). When the ICG/sPDMA weight ratio was 4/14, sPDMA@ICG NPs displayed the smallest particle size and the narrowest polydispersity index (PDI) (Fig. 1c). Hence, sPDMA@ICG NPs prepared at this weight ratio were used for the following investigation. Under the observation of transmission electron microscope (TEM), sPDMA@ICG NPs had a regularly spherical shape (Fig. 1d). The diameter of sPDMA@ICG NPs determined using the dynamic light scattering method was 206 nm and the zeta potential was about + 18.4 mV (Fig. 1e). Through detecting the unloaded ICG, the loading content and encapsulation efficiency of ICG in sPDMA@ICG were further calculated and their values were 19.2% and 86.5%, respectively. Within 5 days, the size and size distribution of sPDMA@ICG NPs changed little (Fig. 1f). In this period, free ICG exhibited greatly decreased absorption intensity in the UV–Vis-NIR absorption spectrum (Fig. 1g), but meanwhile the absorption intensity of sPDMA@ICG NPs almost did not change (Fig. 1h). These results demonstrate sPDMA@ICG NPs have better storage stability than free ICG.

### Photothermal and photodynamic performances of sPDMA@ICG NPs

**Fig. 2:**
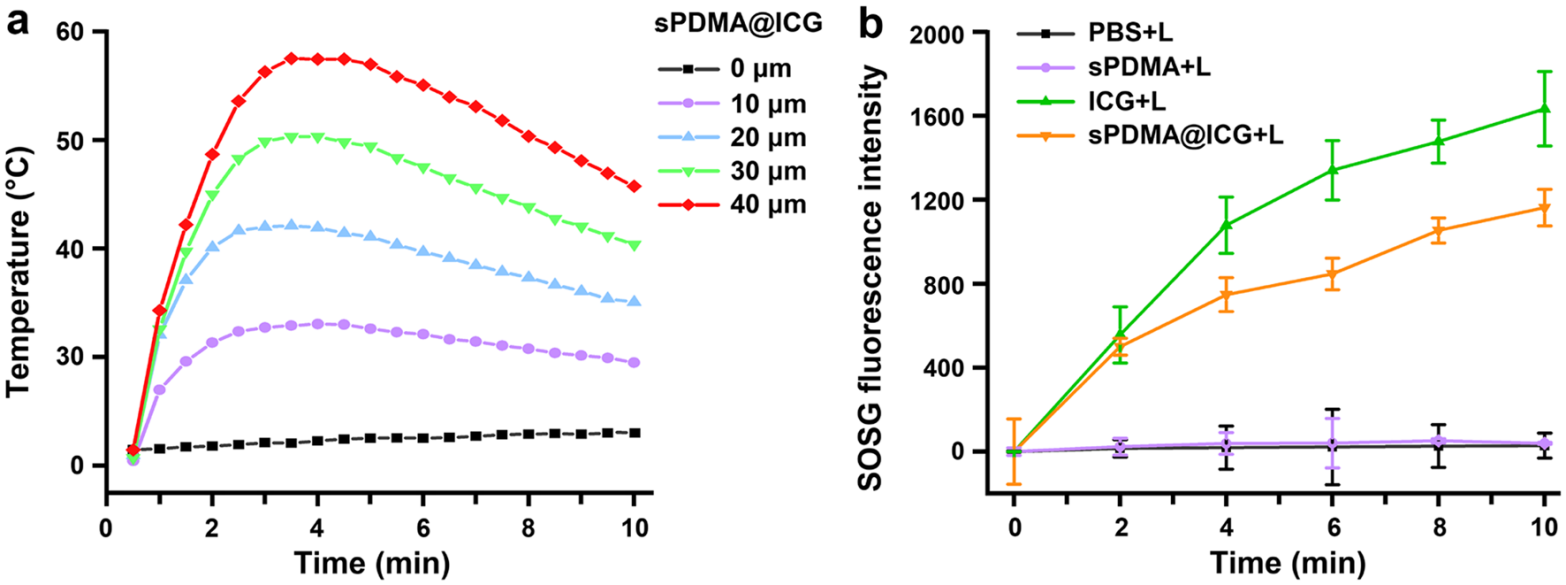
Photothermal and photodynamic performances of the sPDMA@ICG NP solution. **a** Temperature changes of the sPDMA@ICG NPs solutions upon laser irradiation at different ICG concentrations. **b** Fluorescence intensities of SOSG (*λ*_*ex*_ = 504 nm, *λ*_*em*_ = 525 nm) in the solutions of PBS, sPDMA, free ICG and sPDMA@ICG NPs upon laser irradiation (+ L). Here, the ICG concentrations for free ICG and sPDMA@ICG NPs were 10 μg/mL. In the above experiments, the laser irradiation was carried at 808 nm at 2 W/cm^2^ for 10 min

ICG is a photosensitizer with both photothermal and photodynamic performances. After assembled with sPDMA to form PDMA@ICG NPs, the photothermal and photodynamic performances of ICG were further investigated and the results are shown in Fig. 2. After irradiation with an 808 nm laser at 2 W/cm^2^, sPDMA@ICG NPs showed significant temperature elevations in an ICG concentration-dependent manner and the temperatures of their solutions rapidly increased within 2 min (Fig. 2a). Moreover, sPDMA@ICG NPs had slightly higher photothermal efficiency than that of free ICG at the ICG concentration of 35 μg/mL and their solution temperature increased from 22 to 55 °C (Additional file 1: Fig. S4A, B). It thus can be deduced that sPDMA@ICG NPs have good photothermal property. The photodynamic performance of sPDMA@ICG NPs was next evaluated by detecting the ROS generation using a green fluorescence probe, Singlet Oxygen Sensor Green (SOSG). The fluorescence intensity of SOSG at 525 nm for free ICG and sPDMA@ICG NPs (10 μg/mL ICG) both gradually increased with the increase of irradiation time, while it was not observed in phosphate buffered saline (PBS) or sPDMA (Fig. 2b). These results confirmed the significant photodynamic performances of ICG and sPDMA@ICG NPs. By comparison, sPDMA@ICG NPs showed an obviously weaker photodynamic performance, which may be owing to the change of light absorption property of ICG after self-assembly with sPDMA.

### Bacterial surface adsorption and outer membrane penetration of sPDMA@ICG NPs

**Fig. 3:**
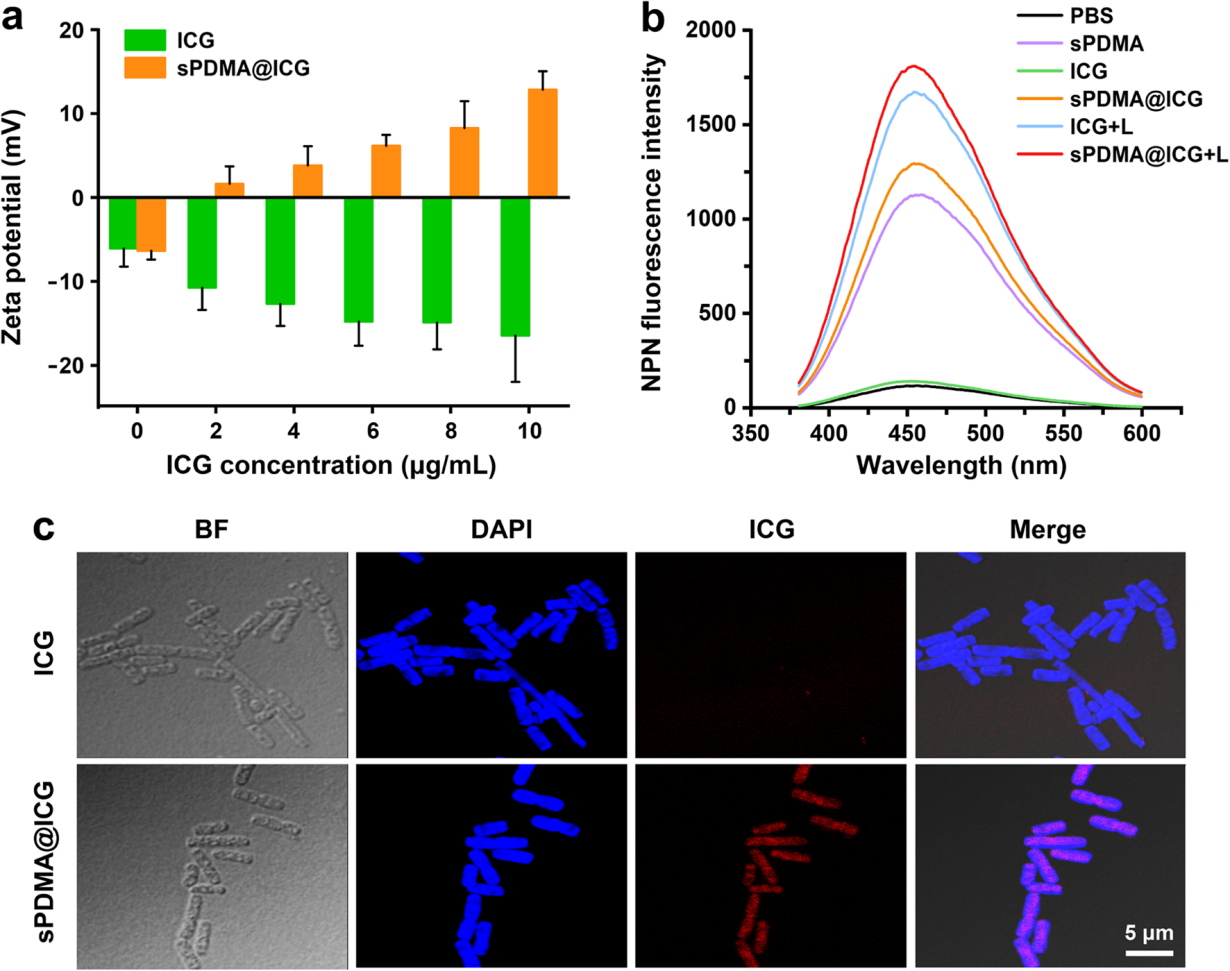
Adsorption and penetration abilities of sPDMA@ICG NPs towards Pg. **a** Zeta potentials of bacteria incubated with free ICG and sPDMA@ICG NPs for 3 h at different ICG concentrations. **b** Fluorescence emission spectra of NPN in bacteria suspensions processed with PBS, sPDMA, free ICG with and without laser irradiation, and sPDMA@ICG NPs with and without laser irradiation. Here, the laser irradiation (808 nm, 2 W/cm^2^ and 5 min) was carried at 3 h after incubation. **c** Confocal images of bacteria with incubation of free ICG and sPDMA@ICG NPs for 3 h. Blue and red fluorescence represent DAPI and ICG, respectively

ICG is negatively charged and water soluble, hence it is difficult for it to pass through the bacterial cell membrane that is also negatively charged and characterized by lipid bimolecular structure. In this study, sPDMA@ICG NPs were prepared from self-assembly of ICG and sPDMA in aqueous solution, hoping to promote the adsorption and penetration of ICG in bacterial cells by taking advantage of the high positive charge density of sPDMA. The surface adsorption of sPDMA@ICG NPs towards Pg, a Gram-negative bacterium that is directly associated with periodontitis, was firstly evaluated through monitoring the change of bacterial surface charge property after 3-h incubation. As shown in Fig. 3a, the negative charges on the surface of Pg incubated with sPDMA@ICG NPs were neutralized continuously, reflecting in the change of zeta potential from negative to positive and the further enhanced zeta potential value as the ICG concentration increasing. But in the meantime, the zeta potential of Pg incubated with free ICG maintained negative values. The above results demonstrate that sPDMA@ICG NPs can be adsorbed onto the bacterial surfaces via electrostatic interaction.

N-phenyl-1-naphthylamine (NPN) is often applied as a fluorescence probe to detect the penetration and damage of bacterial cell membrane, since it emits weak fluorescence in aqueous solution but strong fluorescence after spontaneously entering the hydrophobic region of cell membranes. Herein, NPN was used as a fluorescence indicator to evaluate the penetration of sPDMA@ICG NPs through the outer membrane of Pg, and the results are shown in Fig. 3b. NPN displayed very strong fluorescence signals in the bacteria after 3-h incubation with sPDMA and sPDMA@ICG NPs. However, no visible increased fluorescence intensity of NPN was observed in the bacteria incubated with free ICG as compared to that incubated with PBS. It thus can be deduced that sPDMA has significant bacterial cell membrane-penetrating ability due to the superhigh density of positive charges on its brush layer. Moreover, upon 808 nm laser irradiation at 2 W/cm^2^ for 5 min, free ICG and sPDMA@ICG NPs further enhanced the permeability of outer membrane of Pg, demonstrating the damage of bacterial membrane caused by the photothermal and photodynamic performances of ICG.

The uptakes of free ICG and sPDMA@ICG NPs in Pg were also compared after 3-h incubation through observing the intracellular fluorescence of ICG. Figure 3c shows the confocal microscopic images of the bacteria with staining of 4',6-diamidino-2-phenylindole dihydrochloride (DAPI). The intensive red fluorescence representing ICG was clearly observed in sPDMA@ICG NP-incubated Pg when the ICG concentration was 10 μg/mL, while almost no fluorescence was observed for free ICG with the same concentration. This indicated that sPDMA@ICG NPs were efficiently accumulated in the bacterial cells after penetrating the cell membranes, and thus would facilitate ICG to exert antibacterial effects through synergistic PTT and PDT.

### In vitro antibacterial effects of sPDMA@ICG NPs with laser irradiation

**Fig. 4:**
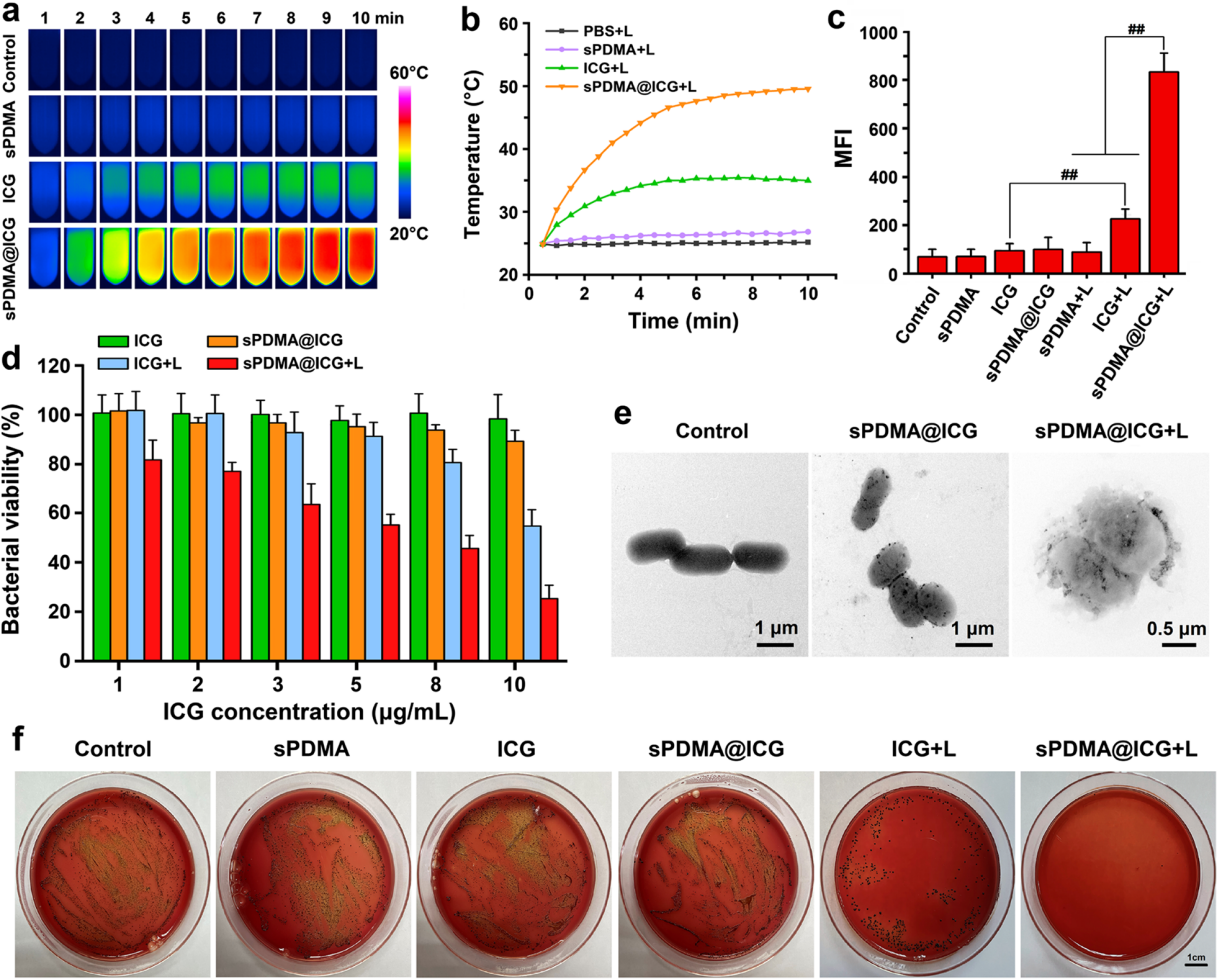
Antibacterial effects of sPDMA@ICG NPs with laser irradiation in Pg. **a** Infrared thermal images and **b** temperature changes of bacterial suspensions incubated with PBS, sPDMA, free ICG and sPDMA@ICG NPs for 3 h, followed by laser irradiation. **c** Mean fluorescence intensities (MFI) of DCF in bacterial suspensions after various processions. **d** Antibacterial activities of free ICG and sPDMA@ICG NPs with and without laser irradiation at different ICG concentrations. **e** TEM images of bacteria after processions of PBS (the control), sPDMA@ICG NPs with and without laser irradiation. **F** Photos of bacterial colonies after various processions. In the experiments for (**a**, **b**, **c**, **e** and **f**), the ICG concentration was 10 μg/mL and the laser irradiation was carried at 808 nm at 2 W/cm^2^ for 10 min. ## indicates *p* < 0.01 for comparison between two groups

Since sPDMA@ICG NPs can effectively deliver ICG into the bacteria cells and exhibit synergistic PTT and PDT performances, we further evaluated the antibacterial effects of sPDMA@ICG NPs. First, the PTT effect of sPDMA@ICG NPs was examined in Pg. After incubation with sample solutions for 3 h, the bacterial suspensions were centrifuged to remove the unbound samples and the precipitations were dispersed in PBS. These bacterial suspensions were irradiated with an 808 nm laser at 2 W/cm^2^ for 10 min, and their temperatures were further recorded using an infrared thermal camera within this period. sPDMA@ICG NPs maintained high photothermal conversion efficiency in the bacterial suspension, which was significantly stronger than free ICG (Fig. 4a, b). After laser irradiation for 5 min, the temperature raised and maintained in the range of 45–50 °C, which was greatly higher than the body temperature. At this temperature range, the oral pathogenic bacteria can be damaged and inhibited effectively. This further proves that sPDMA@ICG NPs efficiently delivered ICG into the bacteria to exert the PTT efficacy against periodontitis.

PDT can induce the generation of ROS that possesses strong cytotoxicity via destroying bacterial lipids, proteins and genes. To evaluate the PDT performance of sPDMA@ICG NPs in Pg, 2’,7’-dichlorodihydrofluorescein diacetate (DCFH-DA) was used as a fluorescent probe to detect the intracellular production of ROS. When a large amount of ROS is produced in the cells, non-fluorescent DCFH-DA can be oxidized into a fluorescent product 2’,7’-dichlorofluorescein (DCF) through a series of reactions. The fluorescence intensity of DCF is related directly to the intracellular level of ROS. Figure 4c compares the fluorescence intensities of DCF in the bacteria. Upon 10 min-laser irradiation at 808 nm at 2 W/cm^2^, free ICG and sPDMA@ICG NPs both induced the notably enhanced fluorescence intensities of DCF, indicating that they exerted the PDT performances to trigger the generations of intracellular ROS. By comparison, sPDMA@ICG NPs showed a significantly more potent PDT performance, inflecting in a higher fluorescence intensity of DCF. This further proves sPDMA@ICG NPs efficiently delivered ICG into the bacteria to exert the PDT efficacy against periodontitis.

The antibacterial activity of sPDMA@ICG NP-mediated PTT and PDT was next evaluated in Pg using the CCK-8 assay. The influence of sPDMA on the bacterial viability was assessed firstly at different concentrations. Results in Additional file 1: Fig. S5 shows that the bacterial viability decreased notably when the sPDMA concentration was larger than 50 μg/mL. Therefore, the concentration of sPDMA below 25 μg/mL was chosen in the following study. Without laser irradiation, the bacterial viabilities in the groups of free ICG and sPDMA@ICG NPs maintained at the high levels in the ICG concentration range of 1–10 μg/mL. After 808 nm laser irradiation at 2 W/cm^2^ for 5 min, free ICG and sPDMA@ICG NPs both inhibited the bacterial growth remarkably, but by comparison, sPDMA@ICG NPs displayed a much higher antibacterial effect (Fig. 4d). Under the TEM observation, the adsorption of sPDMA@ICG NPs was clearly visible on the surfaces of Pg, and after laser irradiation, the bacterial membrane was ruptured and the bacterial cells were disintegrated (Fig. 4e). Colony formation assay was further applied to evaluate the antibacterial activity visibly in Pg. As shown in Fig. 4f, sPDMA@ICG NPs with laser irradiation almost completely inhibited the bacterial growth at the ICG concentration of 10 μg/mL, while the bacteria in the other groups grew in different degrees. These results indicated that sPDMA@ICG NPs exerted synergistic PTT and PTT.

### Destruction effect of sPDMA@ICG NPs with laser irradiation on the plaque biofilm

**Fig. 5:**
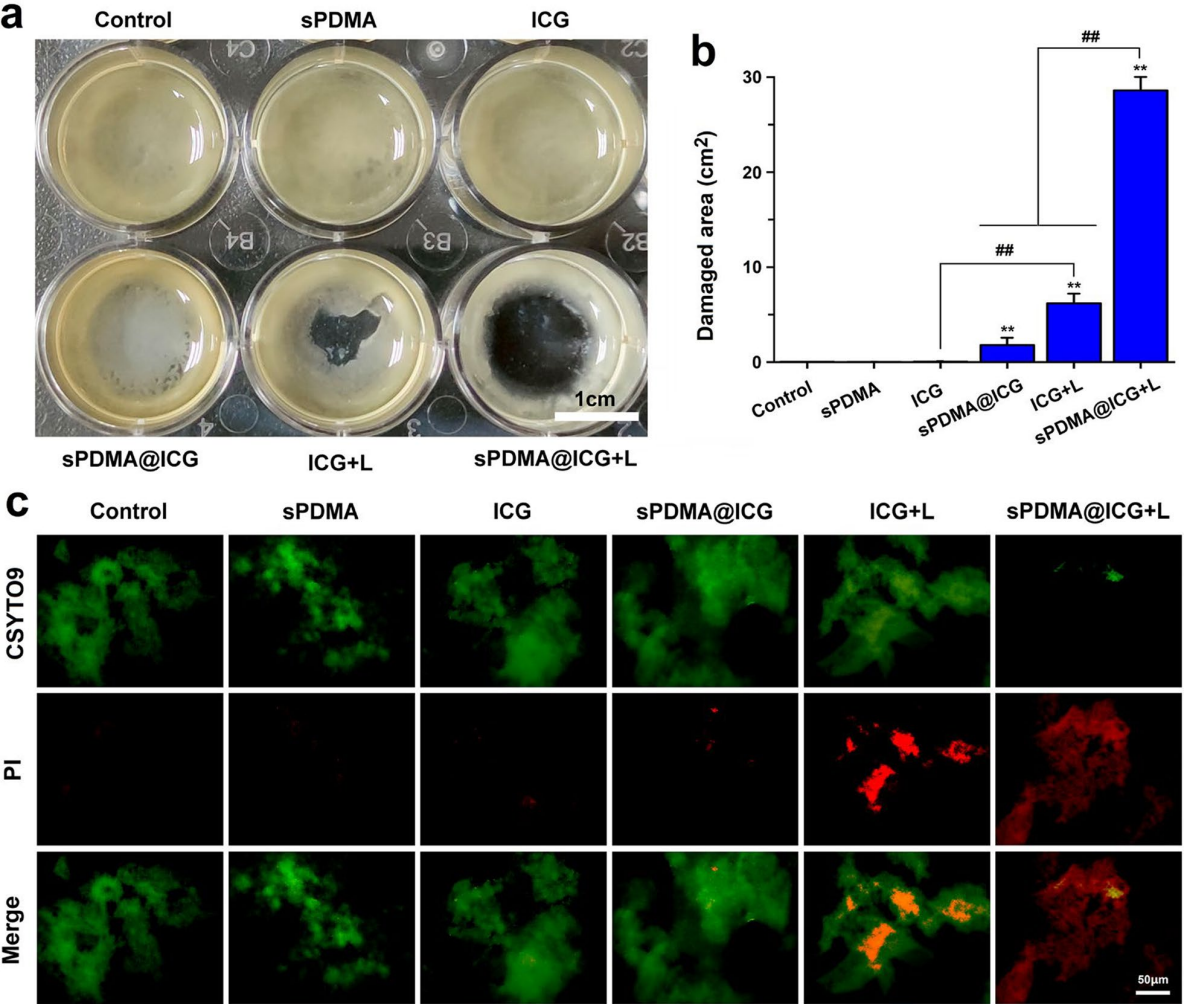
Destruction effect of sPDMA@ICG NPs with laser irradiation on plaque biofilm. **a** Photos of plaque biofilms after various processions and **b** comparison for destructive area analyzed using *Image J*. **c** Fluorescence microscopic images of plaque biofilms stained with the Live/Dead staining kit after various processions. In these above experiments, the plaque biofilms were formed by pathogenic bacteria sourced from periodontitis rats. Here, the plaque biofilms were incubated with sPDMA, ICG and sPDMA@ICG NPs for 3 h, and afterwards, the laser irradiation was carried out at 808 nm at 2 W/cm^2^ for 5 min. **indicates *p* < 0.01 compared to the control; ## indicates *p* < 0.01 for comparison between two groups

Dental plaque, which actually is the same as the bacterial biofilm, has been known to be the basis for the survival of microorganisms. The dental plaque can resist the penetration of antibacterial agents and thus reduce their antibacterial effects [37, 38]. This shows that the destruction of dental plaque will help to antibacterial treatment. Herein, the pathogenic bacteria were extracted from the periodontitis rats and incubated in the liquid culture medium for 3 d to form the plaque biofilm, which was further applied for evaluation of the destruction effect of sPDMA@ICG NP-mediated PTT and PDT. Only upon laser irradiation (808 nm, 2 W/cm^2^ and 5 min), free ICG and sPDMA@ICG NPs remarkably destroyed the plaque biofilms at the ICG concentration of 10 μg/mL (Fig. 5a). Not surprisingly, sPDMA@ICG NPs with laser irradiation exhibited a more potent destruction effect on the plaque biofilm than free ICG with laser irradiation, reflecting in a larger area (2.87 cm^2^) of damaged biofilm (Fig. 5b). These damaged plaque biofilms were further stained with the LIVE/DEAD (SYTO9/PI) BacLight Bacterial Viability Kit to evaluate the antibacterial activity. Under a fluorescence microscope, the live and dead bacteria emitted green and fluorescence separately. Similar to the above results, the bacteria in the group of sPDMA@ICG NPs with laser irradiation were almost completely killed (Fig. 5c). These results suggest that synergistic PTT and PDT mediated via sPDMA@ICG NPs is a promising antibacterial method for periodontitis treatment because of its destruction effect on the plaque biofilm and penetration ability through the bacterial membrane.

### In vivo PTT and PDT performances of sPDMA@ICG NPs

**Fig. 6:**
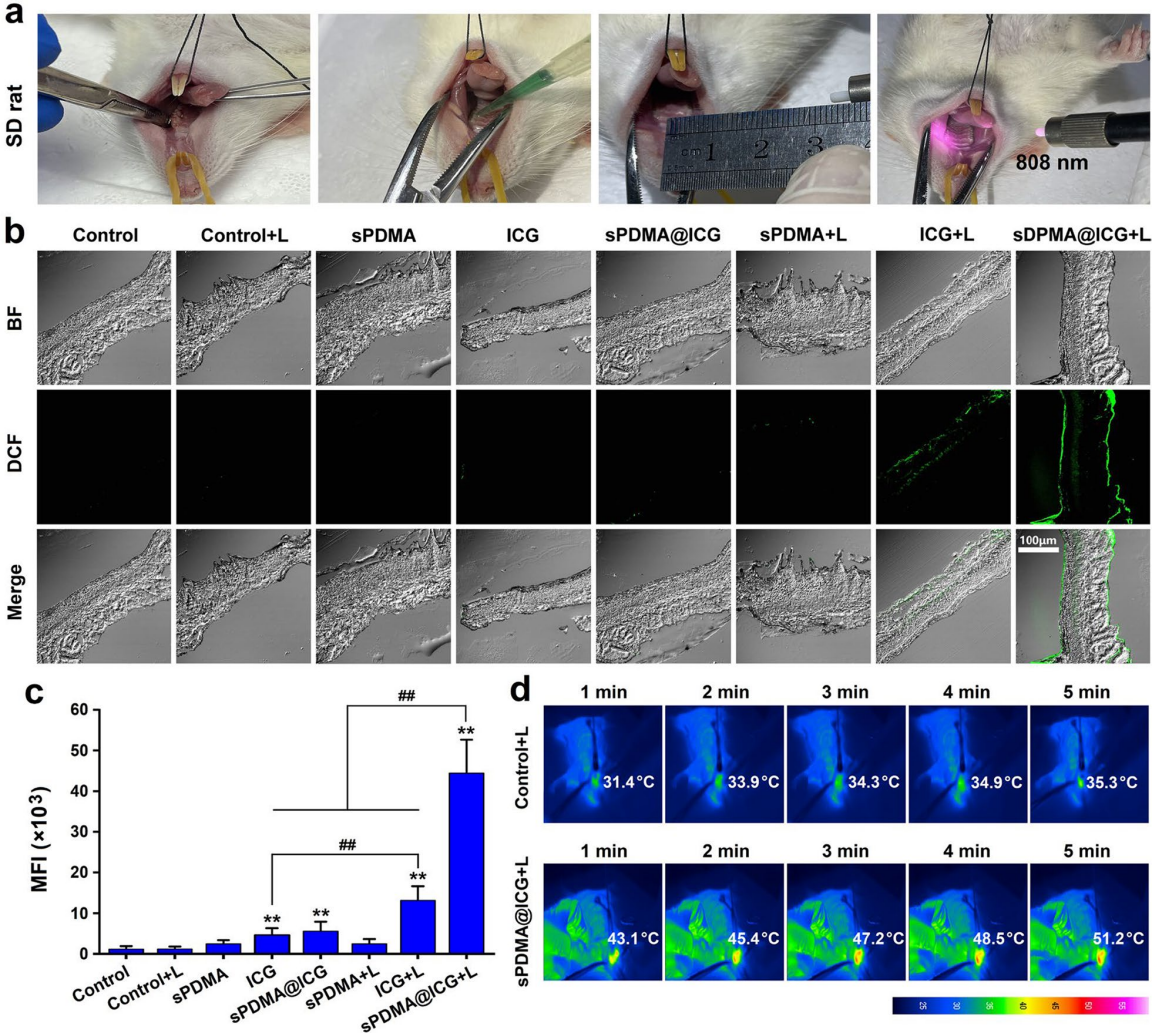
PTT and PDT performances of sPDMA@ICG NPs in periodontitis rats. **a** Photos of periodontitis rat with administration of sPDMA@ICG NPs and laser irradiation. **b** Confocal microscopic images of DCFH-DA stained tissue sections sourced from periodontitis lesions in the rats after treatments of sPDMA, ICG and sPDMA@ICG NPs with or without laser irradiation. **c** Comparison for the mean fluorescence intensities of produced DCF in periodontal tissues. Infrared thermal images of periodontal tissues during laser irradiation after administration of PBS (the control) and sPDMA@ICG NPs. In these experiments, the laser irradiation (808 nm, 2 W/cm^2^, 5 min) was carried out locally at the periodontitis site. **indicates *p* < 0.01 compared to the control ( +); ^##^indicates *p* < 0.01 for comparison between two groups

A periodontitis animal model was constructed in Sprague Dawley (SD) rats according to our previous method [21]. After anesthetization, the rats were ligated with orthodontic steel wires at the gingival sulcus of the left maxillary second molar and then feed with 10% sucrose water for 4 weeks to induce periodontitis. These rats were given the treatments of sPDMA, free ICG and sPDMA@ICG NPs alone and their combination with laser irradiation. Here, 50 μL of sample solutions containing 20 μg/mL of ICG and/or 70 μg/mL of sPDMA were administrated via smear and laser irradiation was carried out at 808 nm at 2 W/cm^2^ for 5 min. Figure 6a shows the photos of periodontitis rat receiving the treatment of sPDMA@ICG NPs and laser irradiation.

DCFH-DA was next used as a fluorescence probe to detect the generation levels of ROS in periodontal tissues at treatment site. Figure 6b shows the confocal microscope images of frozen tissue sections with DCFH-DA staining. The green fluorescence of produced DCF was only observed in the periodontal tissues of the rats receiving the treatments of free ICG and sPDMA@ICG NPs with laser irradiation, demonstrating the generations of ROS induced by these treatments. Figure 6c shows the corresponding quantitative analysis of DCF fluorescence intensities. It was obvious that sPDMA@ICG NPs with laser irradiation induced a much higher level of ROS generation than free ICG with laser irradiation. These results indicated that sPDMA@ICG NPs had a potent PDT performance in periodontitis rats owing to their adhesion and penetration towards the plaque biofilms and bacterial membranes.

Within 5 min of laser irradiation, the temperature changes at periodontal tissues in the rats with administration of PBS (the control) and sPDMA@ICG NPs were also detected using a thermal imaging camera. As shown in Fig. 6d, sPDMA@ICG NPs increased the temperature of periodontal tissue rapidly to 43.1 °C within 1 min of laser irradiation and 51.2 °C within 5 min of laser irradiation. According to an investigation we previously reported [39], this temperature will not bring about the serious damages to the normal tissues. On the contrary, the temperature of periodontal tissue in the control rat did not increase significantly within this period of laser irradiation. These results indicated that sPDMA@ICG NPs also had a potent PTT performance in periodontitis rats.

### In vivo anti-periodontitis effects of sPDMA@ICG NPs with laser irradiation

**Fig. 7:**
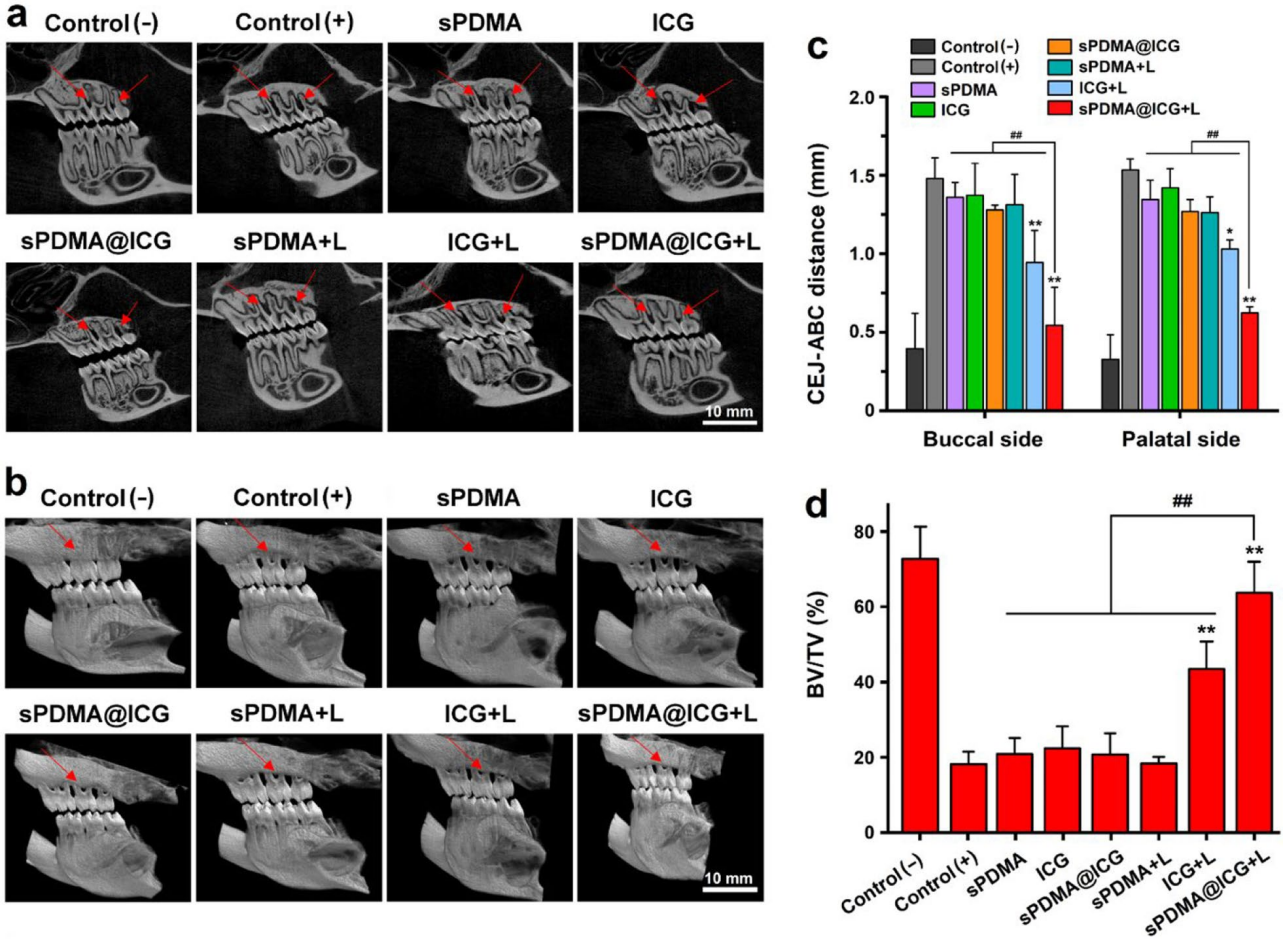
Inhibitory effect of sPDMA@ICG NPs with laser irradiation on the alveolar bone resorption in periodontitis rats. **a** Cross-sectional and **b** three-dimensional micro-CT images of the alveolar bones in the control rats (negative and positive) and the rats receiving the treatments of sPDMA, free ICG, sPDMA@ICG NPs with and without laser irradiation. **c** CEJ-ABC distance on the buccal and palatal sides and **d** BV/TV calculated from micro-CT results. * and **indicate *P* < 0.05 and *P* < 0.01 compared separately to the control ( +); ^*##*^indicates *p* < 0.01 for comparison between two groups

Periodontitis rats were used to evaluate the in vivo anti-periodontitis effects of synergistic PTT and PDT mediated by sPDMA@ICG NPs. The treatments including sPDMA, free ICG, sPDMA@ICG NPs alone and their combination with laser irradiation as described above were carried out once a week for consecutive 3 weeks. Afterwards, the resorption of alveolar bones in these treated rats was analyzed using the microcomputed tomography (micro-CT). The three-dimensional digital and tomographic images of alveolar bones were separately reconstructed with *CTVox* software and *DataViewer* software, shown in Fig. 7a, b. Compared to the negative (−) control rat, the alveolar bone resorption was very obvious in the positive ( +) control rat, confirming the success construct of the periodontitis rat model. Upon laser irradiation, free ICG and sPDMA@ICG NPs both remarkably inhibited the alveolar bone resorption in periodontitis rats. By comparison, sPDMA@ICG NPs with laser irradiation exhibited a much stronger inhibitory effect on the alveolar bone resorption, and the treated rats had the alveolar bone heights similar to that of the control (−) rats. The distance between alveolar bone crest (ABC) and cement enamel junction (CEJ) was determined by the three points from the mesial to distal root surface of the second molars. Bone volume (BV), tissue volume (TV) and their ratios (BV/TV) around the ligated molars were further calculated by using *CTAn* software. Results in Fig. 7c, d showed that the rats receiving the treatment of sPDMA@ICG NPs with laser irradiation had the shortest distance of CEJ-ABC and the highest of BV/TV. These results were basically consistent with the in vitro antibacterial results, confirming that sPDMA@ICG NPs can efficiently prevent the alveolar bone resorption through exerting the PTT and PDT performances.

**Fig. 8:**
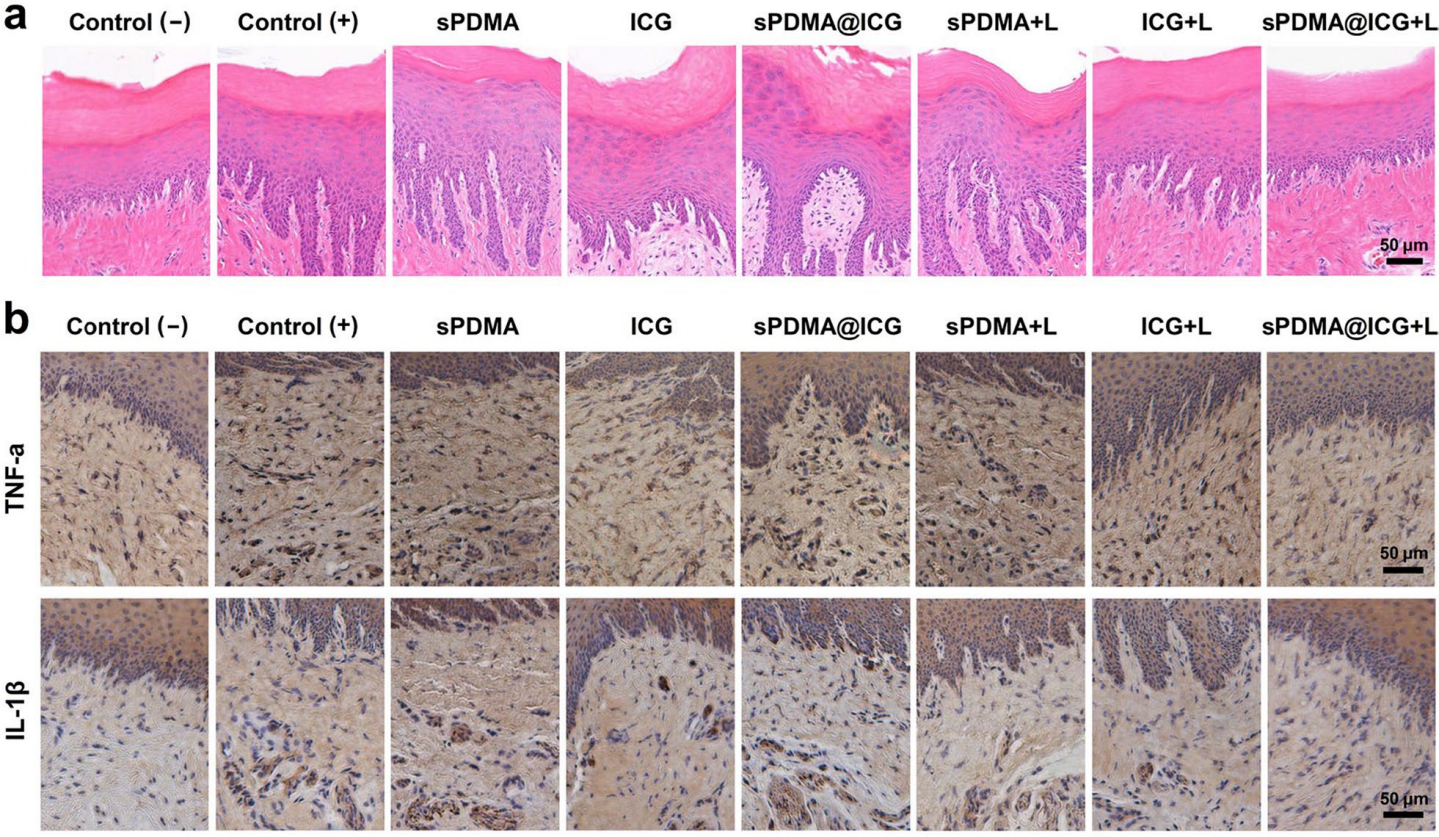
Microscopic images of tissue sections with staining of H&E (**a**), TNF-α and IL-1β antibodies **b** from the control rats (negative and positive) and the rats receiving the treatments of sPDMA, free ICG, sPDMA@ICG NPs with and without laser irradiation

When all treatments were completed, the rats were euthanized and their periodontitis lesions (palatal gingiva and surrounding mucosa) were removed from the left maxillary second molar for histopathological examination. The microscopic images of tissue sections stained with hematoxylin and eosin (H&E) are shown in Fig. 8a. Different degrees of inflammatory responses (incomplete epithelium, epithelial hyperplasia, irregular arrangement of basal cells, and infiltration of inflammatory cells) were observed in the control( +) rats and the rats receiving the treatments of sPDMA with and without laser irradiation, free ICG and sPDMA@ICG NPs. However, these inflammatory responses were greatly ameliorated in the rats treated with free ICG and sPDMA@ICG NPs after laser irradiation. More importantly, sPDMA@ICG NPs with laser irradiation almost completely prevented the progress of periodontitis, which was manifested in the normal morphological and closely arranged epithelial cells as well as no obvious inflammatory changes in lamina propria.

As two of the inflammatory factors, tumor necrosis factor-α (TNF-α) and interleukin-1β (IL-1β) play the important roles in the regulation of local inflammatory responses in periodontitis [40]. TNF-α can induce the inflammation, promote the secretion of matrix metalloproteins, activate the osteoclasts, and destroy the periodontal tissue [41, 42]. IL-1β can recruit and activate the neutrophile granulocytes, improve the release of inflammatory mediators, and decompose the connective tissue [43, 44]. These inflammatory factors will be convenient for pathogenic bacteria and their toxic metabolites to invade deep tissues and promote the occurrence and development of periodontitis [45]. Therefore, we further evaluated the protein levels of TNF-α and IL-1β in tissues by immumohistochemical staining. As can be seen in Fig. 8b, c, sPDMA@ICG NPs with laser irradiation evidently reduced the protein levels of these two inflammatory factors as compared to the control ( +) and the other treatments. It thus can be deduced that synergistic PTT and PDT mediated by sPDMA@ICG NPs relieved the inflammatory responses in periodontitis rats.

## Conclusions

In this work, a nanosystem was developed through self-assembling of ICG and sPDMA in aqueous media for synergistic PTT and PDT against periodontitis. As-prepared sPDMA@ICG NPs were regularly spherical in shape, and their particle size and zeta potential were approximately 206 nm and + 18.4 mV, respectively. Benefiting from the polycationic brush architecture of sPDMA, sPDMA@ICG NPs showed excellent adsorption and penetration abilities for Pg and successfully delivered ICG into the bacteria cells. Upon 808 nm laser irradiation, sPDMA@ICG NPs showed synergistic PTT and PDT performances, and efficiently inhibited the in vitro growth of Pg. In periodontitis rats, sPDMA@ICG NPs effectively inhibited the alveolar bone resorption and relieved the inflammatory responses after laser irradiation. Thus, it can be seen that sPDMA@ICG NPs will be a promising nano-photosensitizer for synergistic PTT and PDT for antibacterial and periodontitis treatments in clinic.

## Supplementary Information

**Additional file 1. **Synthesis methods of CD-Br and sPDMA. In vitro and in vivo experiment methods. **Figure S1.** Synthesis route of sPDMA polycationic brush. **Figure S2.** Chemical structure and ^1^HNMR spectrum of CD-Br in DMSO-d6. **Figure S3.** UV–Vis-NIR absorption spectra of ICG, sPDMA and sPDMA@ICG NPs. **Figure S4.** IR thermal images **a** and temperature changes **b** of solutions containing sPDMA, ICG and sPDMA@ICG NPs during 5 min of 808 nm laser irradiation at 2 W/cm^2^. **Figure S5.** Cytotoxicity of sPDMA at different concentrations in Pg.

## Data Availability

All data generated or analyzed during this study are included in this article and its additional information file.
