# Supplementary material for: Self-assembled nanoparticles containing photosensitizer and polycationic brush for synergistic photothermal and photodynamic therapy against periodontitis
Source: J Nanobiotechnology. 2021 Dec 11;19:413. doi: 10.1186/s12951-021-01114-w (PMC8665613; doi:10.1186/s12951-021-01114-w)
Supplement: Supplementary file 1 — Additional file 1. Synthesis methods of CD-Br and sPDMA. In vitro and in vivo experiment methods. Figure S1. Synthesis route of sPDMA polycationic brush. Figure S2. Chemical structure and 1HNMR spectrum of CD-Br in DMSO-d6. Figure S3. UV–Vis-NIR absorption spectra of ICG, sPDMA and sPDMA@ICG NPs. Figure S4. IR thermal images a and temperature changes b of solutions containing sPDMA, ICG and sPDMA@ICG NPs during 5 min of 808 nm laser irradiation at 2 W/cm2. Figure S5. Cytotoxicity of sPDMA at different concentrations in Pg. [file 12951_2021_1114_MOESM1_ESM.doc]

**Additional file 1**

**Self-assembled nanoparticles containing photosensitizer and polycationic brush for synergistic photothermal and photodynamic therapy against periodontitis**

Enyu Shi^1†^, Liya Bai^2†^, Lujia Mao^1^, Hanping Wang^1^, Xiaoying Yang^2^, Yinsong Wang^2^, Mingming Zhang^3*^, Changyi Li^1*^, Yue Wang^1*^

^1^ School of Dentistry & Hospital of Stomatology, Tianjin Medical University, Tianjin 300070, China

^2^ Tianjin Key Laboratory of Technologies Enabling Development of Clinical Therapeutics and Diagnostics, School of Pharmacy, Tianjin Medical University, Tianjin 300070, China

^3^ Tianjin Key Laboratory of Biomedical Materials, Institute of Biomedical Engineering, Chinese Academy of Medical Sciences & Peking Union Medical College, Tianjin 300192, China

Correspondence:

*Email address:* [zhangmm@bme.pumc.edu.cn](mailto:zhangmm@bme.pumc.edu.cn) (M. Zhang), [lichangyi@tmu.edu.cn](mailto:lichangyi@tmu.edu.cn) (C. Li), and [wangyue1@tmu.edu.cn](mailto:wangyue1@tmu.edu.cn) (Y. Wang)

^†^ Enyu Shi and Liya Bai contributed equally to this work.

**Supporting experimental methods**

**Synthesis of bromo-substituted β-cyclodextrin (CD-Br)**

β-CD (1.135 g, 1 mmol) was dissolved in 7 mL of anhydrous NMP and cooled to 0°C. BiBB (0.742 mL, 6 mmol) in 1 mL of anhydrous NMP was dropwised. After reaction at 0°C for 4 h and 25°C for another 44 h, 50 mL of dichloromethane was added into this mixture. To remove unreacted substances, the organic solution was washed with saturated sodium bicarbonate solution and deionized water respectively for three times. The organic layer was collected, dried over anhydrous MgSO_4_, concentrated via rotary evaporation, and precipitated in n-hexane. The precipitate was vacuum dried to obtain CD-Br. The ^1^H NMR spectrum of CD-Br was recorded on a UNITY-plus 400M spectrometer (Varian, Palo Alto, CA, USA).

**Synthesis of sPDMA polycationic brush**

sPDMA was synthesized via atom transfer radical polymerization (ATRP) of DMA monomer using CD-Br as an initiator. In detail, CuCl (50.49 mg, 0.51 mmol), CuCl_2_ (20.57 mg, 0.153 mmol) and Bpy (207.11 mg, 1.326 mmol) were dissolved in 8 mL of acetone/water (95/5, v/v) and degassed with three freeze-pump-thaw cycles. CD-Br (264.46 mg, 0.12 mmol) and DMA (4.3 mL, 25.5 mmol) were dissolved in 12 mL of acetone/water (95/5, v/v) and then mixed with the above solution under an argon atmosphere. The mixture was degassed with another two freeze-pump-thaw cycles and further reacted under stirring for 20 h at 35°C. The reactant was transferred into a dialysis bag (MWCO 7000 Da) and purified through dialysis against deionized water for 2 d. The dialysate was lyophilized to obtain sPDMA and further characterized by ^1^H NMR spectrum.

**Adsorption evaluation of sPDMA@ICG nanoparticles on bacterial surfaces**

The positively charged sPDMA@ICG nanoparticles will spontaneously adhere to the negatively charged surfaces of bacteria via charge interactions. Hence, we can evaluate the adsorption of these nanoparticles through detecting the changes of bacterial surface charges. Typically, the suspensions of *P. gingivalis* were diluted to 1×10^4^ CFU/mL and followed by incubation separately with ICG and sPDMA@ICG nanoparticles for 1 h. The concentration of ICG was increased from 0 to 10 μg/L, and correspondingly the concentration of sPDMA ranged from 0 to 35 μg/mL. Afterwards, these bacteria were centrifuged and washed with deionized water to remove the unabsorbed agents, and finally resuspended in 1 mL of deionized water to detect their Zeta potentials.

By using the fluorescence of ICG itself, we detected visibly the adsorption of sPDMA@ICG nanoparticles on *P. gingivalis* after incubation. Specifically, the bacteria suspensions were diluted to 1×10^4^ CFU/mL and cultured for 15 h, and next incubated with ICG and sPDMA@ICG nanoparticles for 3 h at the ICG and sPDMA concentrations of 10 and 35 μg/mL, respectively. After that, the bacteria were fixed with 4% polyformaldehyde, stained with DAPI and finally observed under a FV1000 confocal laser scanning microscope (Olympus, Tokyo, Japan).

**Permeability measurement of bacterial outer membrane**

The fluorescence probe N-phenyl-1-naphthylamine (NPN) was used to detect the penetration changes of bacterial outer membrane after interaction with sPDMA@ICG nanoparticles. The suspensions of *P. gingivalis* with a concentration of 1×10^8^ CFU/mL were incubated separately with PBS (the control), ICG, sPDMA, and sPDMA@ICG nanoparticles for 3 h at the concentrations of ICG and sPDMA of 10 and 35 μg/mL, respectively. Next, the bacteria in the laser irradiation groups were exposed to an 808 nm for 5 min at a power density of 2 W/cm^2^. After that, NPN was added to these bacteria both with and without laser irradiation at a concentration of 20 μmol/L and followed by incubation for 1 h. Finally, the fluorescence emission spectra of NPN in these bacteria were recorded under a F-7000 fluorescence spectrophotometer (Shimadzu, Tokyo, Japan) at the excitation wavelength of 350 nm.

**In vitro evaluation of photothermal performance**

We firstly evaluated the photothermal performances of sPDMA@ICG nanoparticles in deionized solutions at different ICG concentrations (10, 20, 30, and 40 μg/mL) during 10 min of 808 nm laser irradiation with a power density of 2 W/cm^2^ by monitoring their temperature changes using an IR thermal imaging camera (FLIR Corporation, USA). The photothermal performances of sPDMA, ICG and sPDMA@ICG nanoparticles were compared meanwhile through detecting their temperature changes at the ICG and sPDMA concentrations of 35 μg/mL and 1.25 mg/mL, respectively.

Next, we evaluated the photothermal performance of sPDMA@ICG nanoparticles in *P. gingivalis*. The bacteria were diluted into aseptic enzyme-free EP tubes with a volume of 2 mL at a concentration of 1×10^8^ CFU/mL. Then, PBS (as the control), sPDMA, ICG, and sPDMA@ICG nanoparticles were added and the ICG and sPDMA concentration were 10 and 35 μg/mL, respectively. After incubation for 3 h, the bacterial suspensions were centrifuged for 10 min at 3000 rpm and the bacteria were washed with PBS, followed by dispersion in 1 mL of PBS. These bacterial suspensions were exposed to an 808 nm laser with a power density of 2 W/cm^2^ for 10 min and their temperatures were scanned using an IR thermal imaging camera (FLIR Corporation, USA).

**In vitro evaluation of photodynamic performance**

The photodynamic performance of sPDMA@ICG nanoparticles was assessed by measuring the ROS generation after laser irradiation. SOSG was firstly used as a fluorescence probe to evaluate the ROS generation levels in PBS (the control) and the solutions containing sPDMA, ICG and sPDMA@ICG nanoparticles with the ICG and sPDMA concentrations of 10 and 35 μg/mL according to the manufacture's instruction. Briefly, these sample solutions were mixed with 1 μL of SOSG reagent and then irradiated with an 808 nm laser at a power density of 2 W/cm^2^ for 0, 2, 4, 6, 8, and 10 min, respectively. After that, the fluorescence intensities of SOSG in these sample solutions were detected using a fluorescence spectrophotometer at the excitation and emission wavelengths of 504 and 525 nm, respectively.

DCFH-DA was further used as a cellular ROS fluorescence probe to evaluate the photodynamic performance of sPDMA@ICG nanoparticles in *P. gingivalis*. Typically, the bacteria were diluted to 1×10^8^ CFU/mL and then incubated separately with PBS (the control), sPDMA, ICG, sPDMA@ICG nanoparticles for 3 h at the ICG and sPDMA concentrations of 10 and 35 μg/mL. After that, the bacteria in the laser irradiation groups were irradiated with an 808 nm laser for 10 min at 2 W/cm^2^. DCFH-DA was next added into all bacteria suspensions at a ratio of 1:2000 and followed by incubation for another 30 min. After centrifugation at 3000 rpm for 10 min, the bacteria were washed and resuspended in PBS, and afterwards the fluorescence signals were detected under a fluorescence spectrophotometer at the excitation and emission wavelengths of 488 nm and 525 nm, respectively.

**In vitro assessment of antibacterial activity**

CCK8 assay was applied to assess the antibacterial activity of synergistic PTT and PDT treatment mediated by sPDMA@ICG nanoparticles. Typically, *P. gingivalis* was adjusted to a bacterial concentration of 1×10^4^ CFU/mL and then seeded into the 96-well plates. After culture overnight, these bacteria were incubated with sPDMA, ICG and sPDMA@ICG nanoparticles for 3 h. Here, the concentrations of ICG were 1, 2, 3, 5, 8, and 10 g/mL, and the concentrations of sPDMA were 3.5, 7, 10.5, 17.5, 28, and 35 μg/mL, correspondingly. Next, the bacteria in the laser irradiation groups were exposed to an 808 nm laser for 5 min at 2 W/cm^2^. After incubation for 3 h, all bacteria both with and without laser irradiation were processed with CCK8 reagent according to the manufacture's instruction, and next, the absorbance of each well was detected at 450 nm using a microplate reader (Multiskan GO, Thermo Fisher Scientific, USA).

Colony formation assay was further used to visibly evaluate the antibacterial activity of sPDMA@ICG nanoparticles mediated synergistic PTT and PDT treatment in *P. gingivalis*. After receiving various treatments as above mentioned, the bacteria were diluted 100 times with culture medium, and then 10 µL of bacterial dispersions were spread onto Columbia blood agar plates containing 0.1% chlorhematin and 0.1% vitamin K1. After culture for 7 d, the bacterial colonies were observed and photographed using a digital camera.

**Plaque biofilm destruction experiment**

The gingival crevicular fluid was taken from the periodontitis rats and cultured in Columbia blood agar plates to form the visible colonies of pathogenic bacteria. These bacteria colonies were picked out and cultured in the liquid culture medium. When the concentration was approximately 1×10^6^ CFU/mL, the bacteria were seeded into the 24-well plates and cultured for 3 d to form the complete plaque biofilms. These plaque biofilms were processed with various treatments as mentioned above and followed by incubation for another 24 h, and then their damages were observed and photographed using a digital camera. In addition, these treated plaque biofilms were further stained with the LIVE/DEAD (SYTO9/PI) BacLight Bacterial Viability Kit according to the manufacturer's protocol and imaged using a fluorescence microscope to observe the antibacterial activity visibly.

**In vivo evaluation of PDT and PTT performances**

DCFH-DA was used as a ROS fluorescence probe to evaluate the PDT performance of sPDMA@ICG nanoparticles *in vivo*. Briefly, periodontitis rats were anesthetized and administrated with 50 μL of sample solutions (sPDMA, ICG and sPDMA@ICG nanoparticles) containing 20 μM of DCFH-DA at the site of periodontitis lesion. Here, the ICG and sPDMA concentrations were 20 and 70 μg/mL, respectively. At 5 min afterwards, the rats received the laser irradiation (808 nm, 2 W/cm^2^ and 5 min) at the site of periodontitis lesion. These rats were euthanized 3 h later, and their palatal gingival as well as mucosal tissues adjacent to the site of laser irradiation were then collected and frozen sectioned. The thus obtained frozen sections were further observed and imaged under a confocal microscope.

The photothermal performance of sPDMA@ICG nanoparticles *in vivo* was evaluated in periodontitis rats. PBS (the control) and sPDMA@ICG nanoparticles were smeared onto the gingival sulcus of periodontitis rats. At 3 min afterwards, these rats received the laser irradiation as above mentioned, and in the meantime, the temperatures at the site of laser irradiation were recorded using an IR thermal imaging camera every 30 s.

**In vivo evaluation of anti-periodontitis efficacy**

SD rats were randomly divided into 8 groups (negative control, positive control, and treatment groups of sPDMA with and without laser irradiation, ICG with and without laser irradiation, and sPDMA@ICG nanoparticles with and without laser irradiation) with 3 rats per group. In the negative control group, the orthodontic steel wire was used to ligate the left maxillary second molar of the rats, but removed immediately. In the positive and treatment groups, periodontitis rats were administrated separately with 50 μL of PBS, sPDMA, ICG, and sPDMA@ICG nanoparticles (20 μg/mL ICG and 70 μg/mL sPDMA) at the site of periodontitis lesion. After 3 min, the rats in the laser irradiation groups were processed with laser irradiation as mentioned above. All these treatments were carried out once a week for consecutive 3 weeks.

**Microcomputed tomography (micro-CT) analysis**

When all treatments were completed, the rats were anesthetized and analyzed under a Micro-CT scanner (SkyScan 1276, Bruker, Belgium) to evaluate the alveolar bone resorption. Micro-CT scan was operated at a source voltage of 85 kV, a source current of 200 μA, and a seam thickness of 80 μm. Three-dimensional digital images of alveolar bones were reconstructed by using *CTVox* software and tomographic images of alveolar bones were produced under *DataViewer* software. Bone volume (BV), tissue volume (TV) and their ratios (BV/TV) around the ligated molars were obtained through *CTAn* software. The distance between alveolar bone crest (ABC) and cement enamel junction (CEJ) was determined by the three points from the mesial to distal root surface of the second molars.

**Histopathological and immunohistochemical analysis**

The above rats were euthanized and their periodontitis lesions (palatal gingiva and surrounding mucosa) were removed from the left maxillary second molar for further pathological section. Specific steps included fixation, alcohol gradient dehydration, paraffin embedding, and section. For pathological examination, these obtained sections were stained with H&E and then imaged under an Olympus light microscope. In addition, these sections were also processed with immunohistochemical staining of TNF-α and IL-1β. Here, the rabbit anti-TNF-α antibody (ab6671, Abcam) and anti-IL-1β antibody (ab9722, Abcam) were used as the primary antibodies, and biotinylated goat anti-rabbit IgG was used as the secondary antibody. The stained sections were finally observed and imaged under a microscope.

**Additional file 1 Figures**


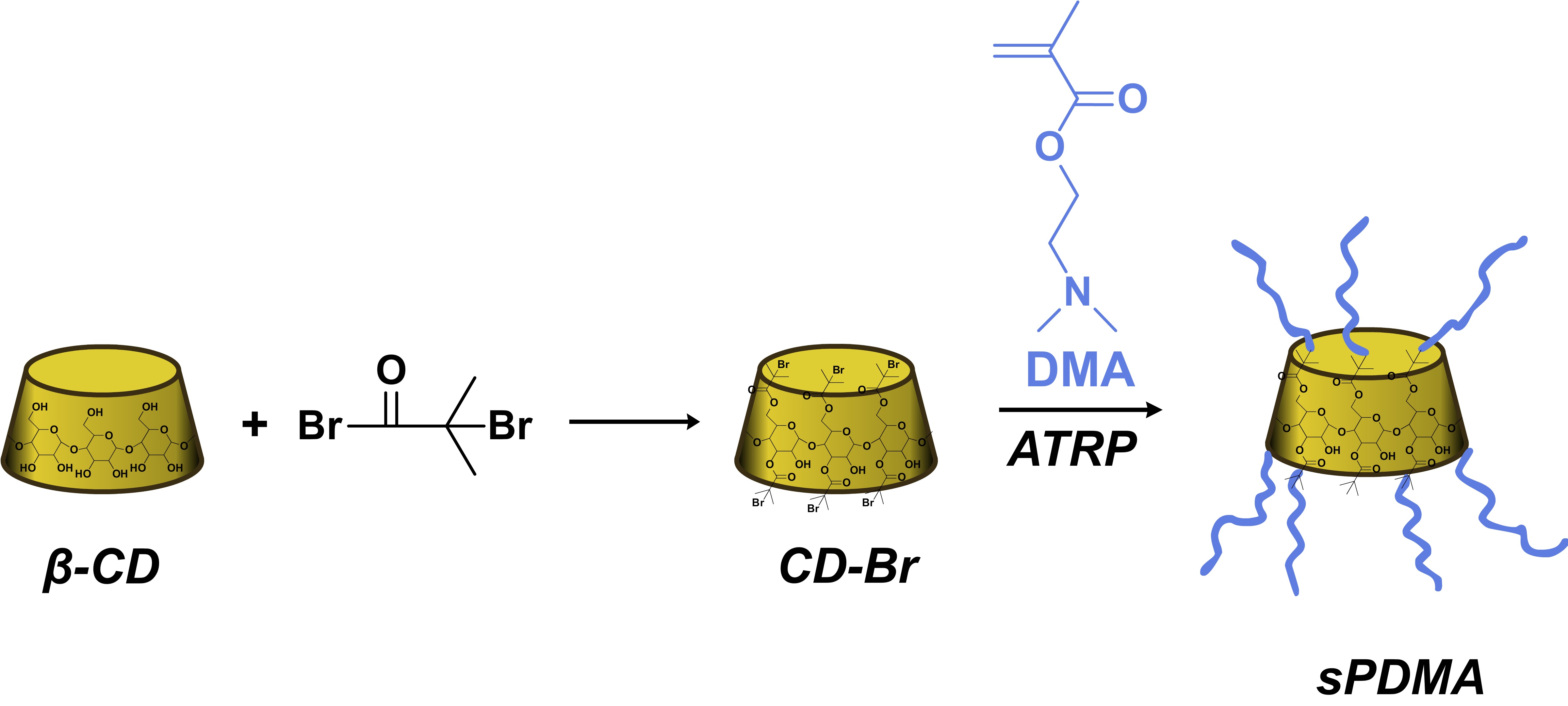


**Fig. S1** Synthesis route of sPDMA polycationic brush.


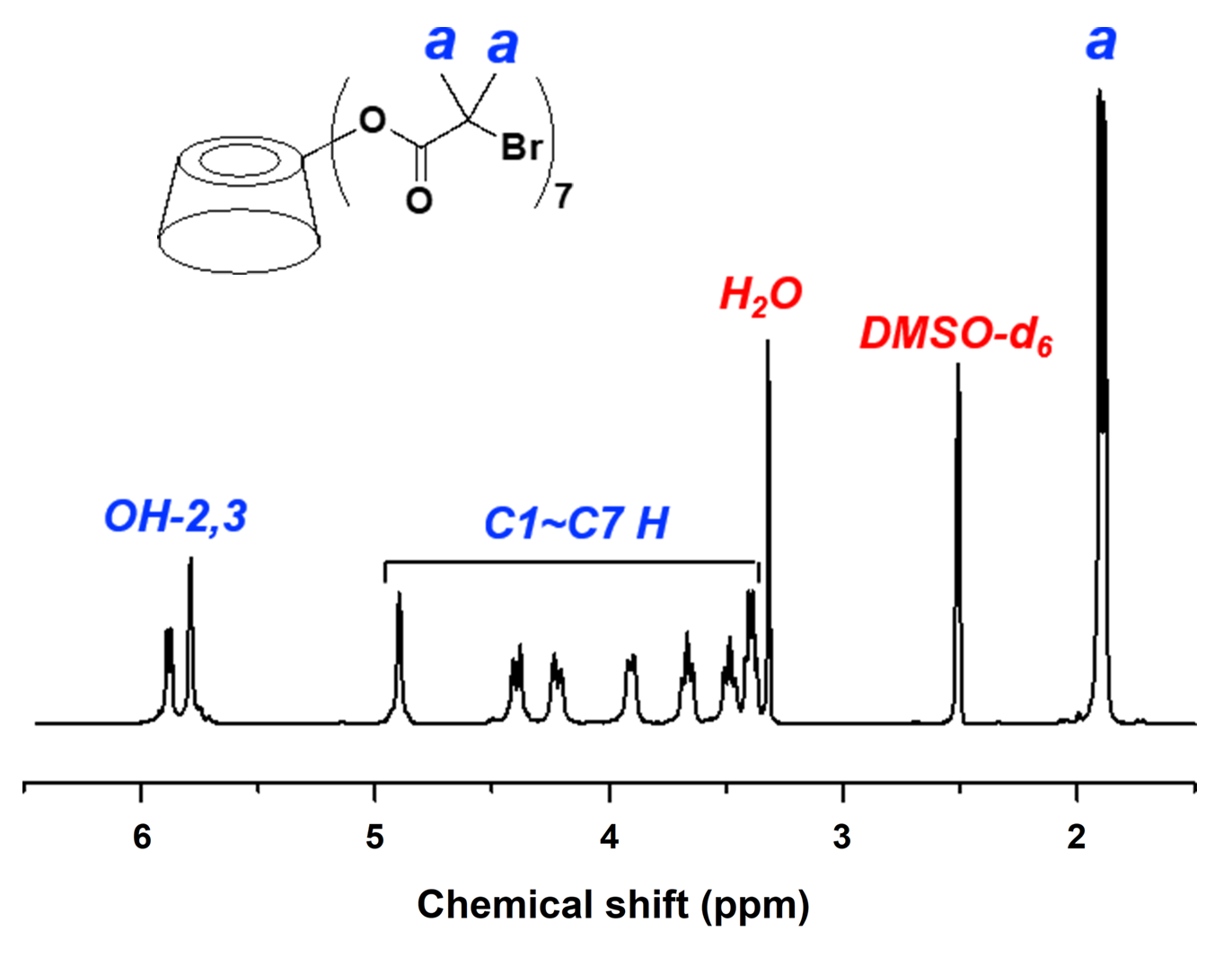


**Fig. S2** Chemical structure and ^1^HNMR spectrum of CD-Br in DMSO-*d_6_*.


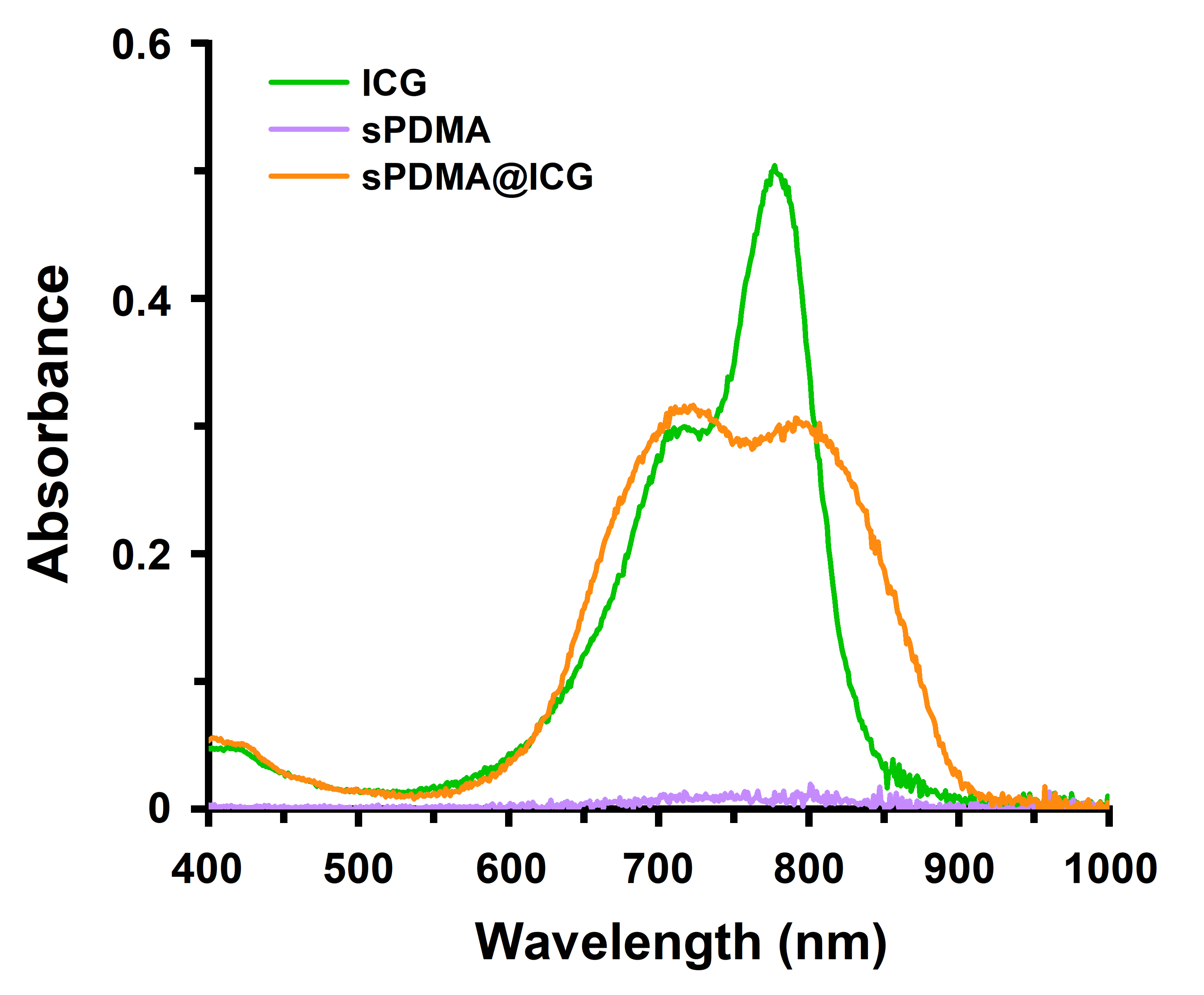


**Fig. S3** UV-Vis-NIR absorption spectra of ICG, sPDMA and sPDMA@ICG nanoparticles. ICG and sPDMA@ICG nanoparticles had the same ICG concentrations, and the ICG/sPDMA weight ratio in sPDMA@ICG nanoparticles was about 4/14.


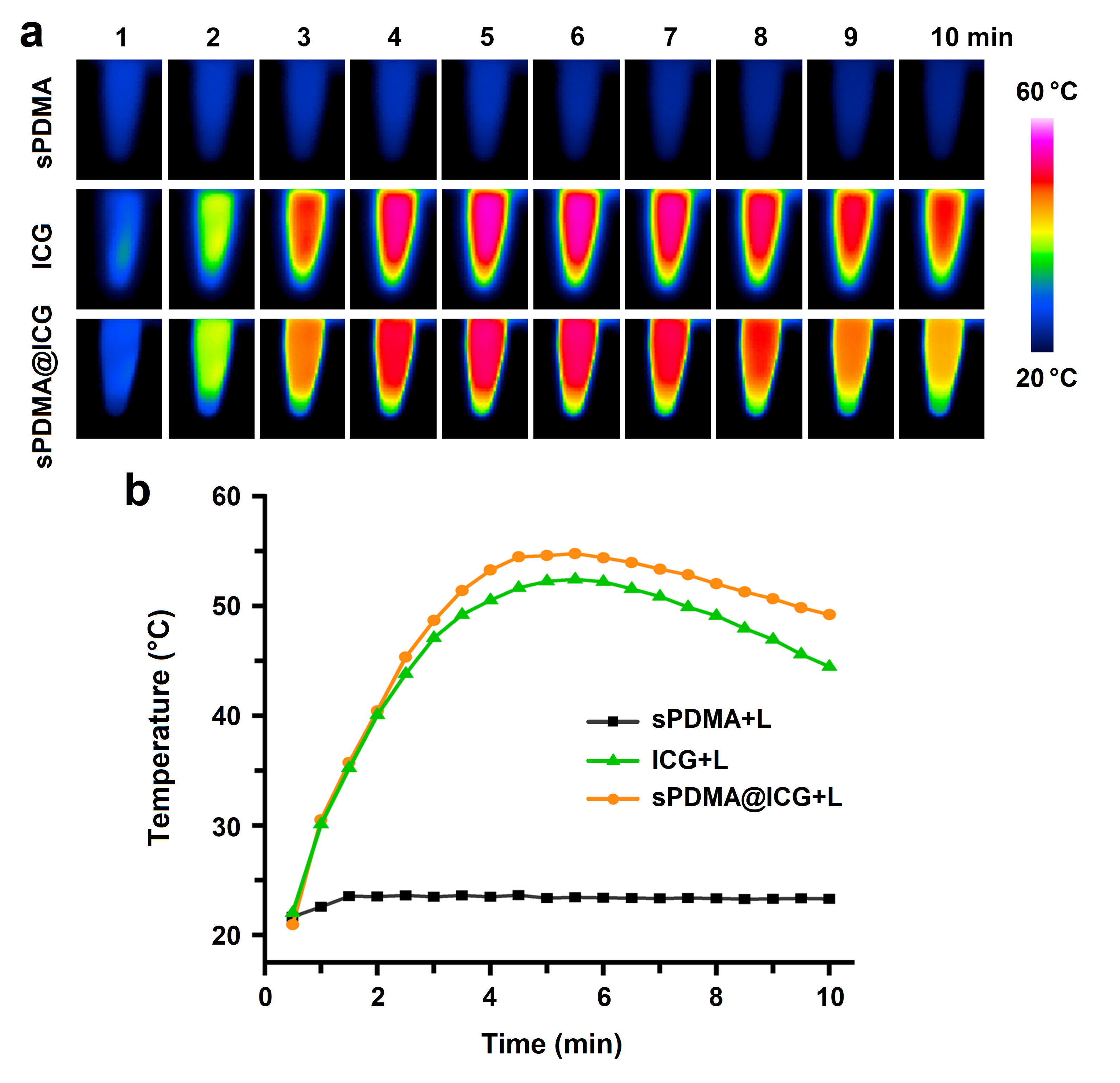


**Fig. S4** IR thermal images (a) and temperature changes (b) of solutions containing sPDMA, ICG and sPDMA@ICG NPs during 5 min of 808 nm laser irradiation at 2 W/cm^2^. The ICG and sPDMA concentrations were 35 μg/mL and 1.25 mg/mL, respectively.

**
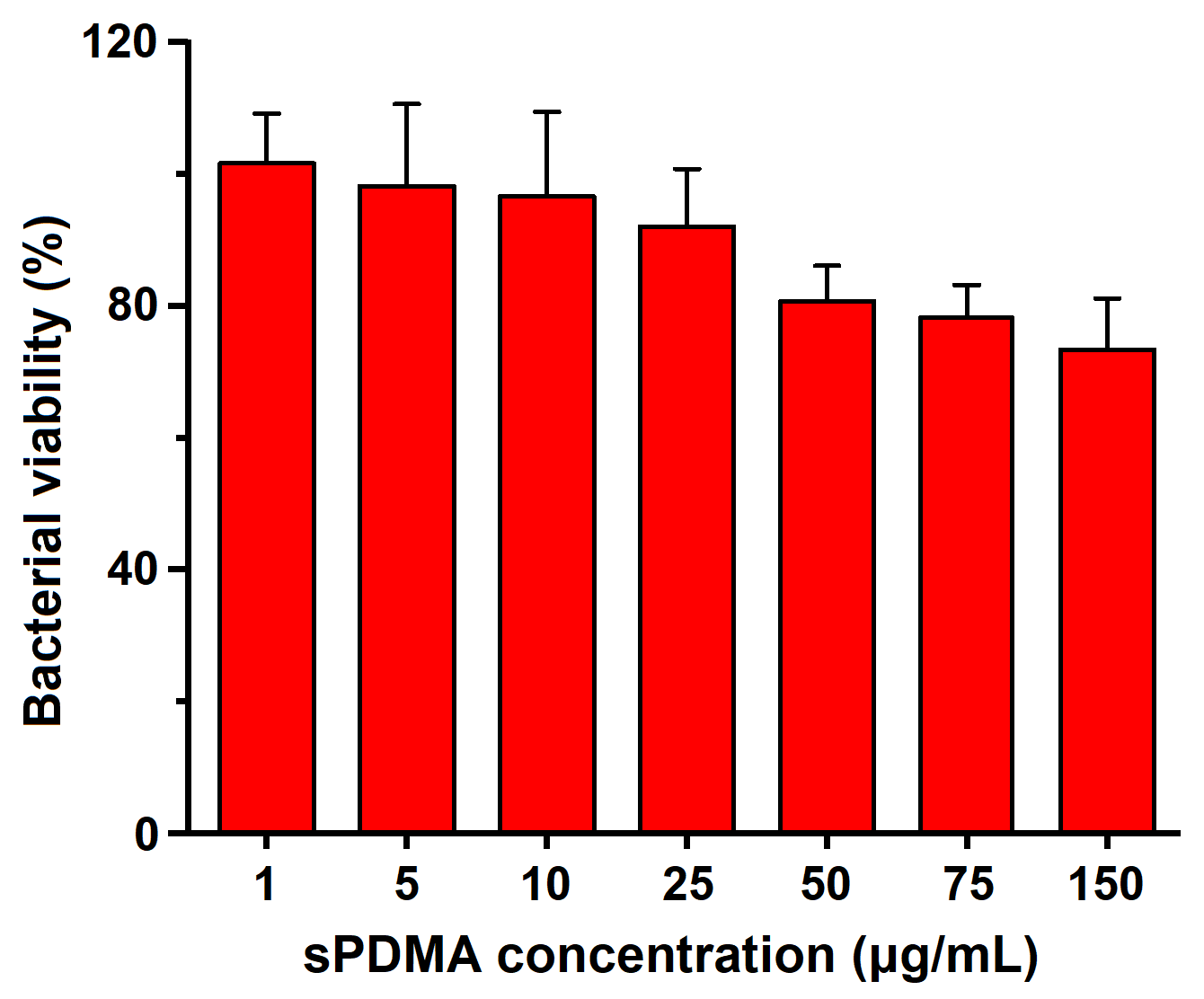
**

**Fig. S5** Cytotoxicity of sPDMA at different concentrations in Pg.
